# Cardiovascular diseases mortality and alcohol control policy in Lithuania: exploring a possible link

**DOI:** 10.1186/s12889-021-12177-7

**Published:** 2021-11-17

**Authors:** Ricardas Radisauskas, Kawon Victoria Kim, Shannon Lange, Vaida Liutkute-Gumarov, Olga Mesceriakova-Veliuliene, Janina Petkeviciene, Mindaugas Stelemekas, Tadas Telksnys, Alexander Tran, Jürgen Rehm

**Affiliations:** 1 Department of Environmental and Occupational Medicine, Faculty of Public Health, Lithuanian University of Health Sciences, Tilzes str. 18, 47181 Kaunas, Lithuania; 2 Institute of Cardiology, Lithuanian University of Health Sciences, Sukileliu av. 15, 50162 Kaunas, Lithuania; 3 Institute for Mental Health Policy Research, Centre for Addiction and Mental Health, 33 Ursula Franklin Street, Toronto, Ontario M5T 2S1 Canada; 4 Dalla Lana School of Public Health, University of Toronto, 155 College Street, Toronto, Ontario M5T 1P8 Canada; 5 Campbell Family Mental Health Research Institute, Centre for Addiction and Mental Health, 33 Russell Street, Toronto, Ontario M5T 2S1 Canada; 6 Health Research Institute, Faculty of Public Health, Lithuanian University of Health Sciences, Tilzes str. 18, 47181 Kaunas, Lithuania; 7 Department of Health Management, Faculty of Public Health, Lithuanian University of Health Sciences, Tilzes str. 18, 47181 Kaunas, Lithuania; 8 Department of Preventive Medicine, Faculty of Public Health, Lithuanian University of Health Sciences, Tilzes str. 18, 47181 Kaunas, Lithuania; 9 Institute of Clinical Psychology and Psychotherapy, Technische Universität Dresden, Chemnitzer str. 46, 01187 Dresden, Germany; 10 Department of Psychiatry, University of Toronto, 250 College Street, Toronto, Ontario M5T 1R8 Canada; 11 Institute of Medical Science, University of Toronto, 1 King’s College Circle, Toronto, Ontario M5S 1A8 Canada; 12 Department of International Health Projects, Institute for Leadership and Health Management, I.M. Sechenov First Moscow State Medical University, Trubetskaya str., 8, b. 2, Moscow, Russian Federation 119992

**Keywords:** Cardiovascular diseases, Mortality, Alcohol consumption, Alcohol control policy, Trends, Sex, Age

## Abstract

**Background:**

Lithuania possesses one of the highest alcohol per capita consumption and has previously implemented alcohol control policies to reduce the alcohol-attributable burden. The aim of this study was to investigate Lithuanian cardiovascular disease (CVD) mortality rate trends between 2001 and 2018 and to explore a possible link between CVD mortality rate and alcohol control policy implementation.

**Methods:**

Lithuanian population mortality and alcohol consumption data for 2001–2018 were obtained from Statistics Lithuania and The State Register of Death Cases and Their Causes, Institute of Hygiene. Sex-specific CVD mortality rates were directly standardized to the European standard population by five-year age groups and categorized according to the ICD-10 codes for all CVDs (I00-I99), ischemic heart disease (IHD) (I20-I25), cerebrovascular diseases (I60-I69) and alcoholic cardiomyopathy (ACM) (I42.6). Joinpoint regression analyses were performed to identify points of inflection to explore their alignment with five selected alcohol policy enactments.

**Results:**

Overall, the 2001–2018 yearly mortality rates for all CVDs significantly decreased on average by − 1.6% (95% CI -2.0, − 1.2%) among men and − 2.1% (95% CI -2.5, − 1.8%) among women. Yearly changes in all CVDs, IHD, cerebrovascular diseases and ACM mortality rates were insignificant prior to their respective critical year points in 2006, 2005, 2008 and 2007, but significantly decreased afterwards by an average of − 2.4% (95% CI -2.7, − 2.0%), − 1.6% (95% CI -2.1, − 1.1%), − 1.2% (95 CI -1.7, − 0.6%) and − 4.5% (95% CI -7.3, − 1.6%) among men, and by − 2.7% (95% CI -3.0, − 2.3%), − 2.0% (95% CI -2.6, − 1.4%), − 1.8% (95% CI 2.4, − 1.3%) and − 6.6% (95% CI -10.7, − 2.2%) among women, respectively. The changes in the mortality rate trends for all CVDs, IHD, cerebrovascular diseases and especially ACM coincided with alcohol policies enacted on the January 1, 2008, January 1, 2009, April 1, 2014 and March 1, 2017.

**Conclusions:**

Yearly mortality rates for all CVDs, IHD, cerebrovascular diseases and ACM have declined in Lithuania between 2001 and 2018, and declining trends were more prominent in women than in men. Among the ICD-10 CVD categories investigated, the points of inflection identified for the ACM mortality rate trend coincided best with the selected alcohol policy enactment dates.

## Background

Following decades of decline globally, cardiovascular disease (CVD) mortality rates have been starting to stagnate in most high-income countries, and even increase in some [1]. In 2019, CVDs were responsible for more than 18.6 million deaths globally, 3.5 million of which were in high income countries [1].

Lithuania has been classified by the World Bank as a high-income country since 2012 but the age-standardized CVD mortality rate per 100,000 people is about 2.6 times greater than that of other high-income countries [2]. In fact, the age-standardized CVD mortality rate is higher in Lithuania than the average for all World Bank income classification groups, as well as the global average [2].

CVD mortality can be mostly attributable to biological risk factors, including arterial hypertension, dyslipidemia and diabetes [3–6], as well as certain health behaviors, such as smoking, unhealthy diet, physical inactivity and alcohol use [7–9]. The observation that Lithuania possesses one of the highest alcohol per capita consumption in tandem with the fact that alcohol use accounts for the highest proportion of CVD deaths and disability-adjusted life years in Europe attests to the association between alcohol use and CVD mortality [10].

Long-term prospective studies have shown that there is a J-shaped association between CVD morbidity and mortality and alcohol use [11–13].

A meta-analysis of 83 prospective studies found that among 599,912 current drinkers, increased alcohol consumption was log-linearly associated with a lower risk of myocardial infarction (Hazard Ratio = 0.94, 95% Confidence Interval (CI): 0.91–0.97) [14]. On the other hand, according to the NHANES cohort, alcohol use increased the risk of coronary artery disease by an average of 14% (Odds Ratio = 1.14, 95% CI: 1.04–1.35) among people under 55 years of age [14]. Heavy drinking, defined as consuming more than 4 drinks on any day or more than 14 drinks per week for men and consuming more than 3 drinks on any day or more than 7 drinks per week for women, is associated with a high risk of death from CVD [10]. Heavy alcohol use, especially among middle-aged men, is a common cause of arterial hypertension, non-ischemic dilated cardiomyopathy (alcoholic cardiomyopathy), atrial fibrillation, and stroke (both ischemic and hemorrhagic) [12, 15].

There is a vast amount of literature that suggests alcohol control policy interventions, such as restrictions on the physical availability of alcohol, an increase of excise tax on alcoholic beverages, and alcohol advertising restrictions can reduce not only all-cause mortality [16], but also specifically CVD mortality [17]. Alcohol control policy is often in the spotlight of political and public debate in Lithuania. In fact, the Alcohol Control Law is one of the most frequently amended legal acts in Lithuanian legislative history with around 63 amendments since 1995 [18]. Not surprisingly, there have been fluctuations between a more conservative stance on alcohol and liberalization, which is suspected to be reflected in the alcohol-related harm trends in Lithuania.

The aim of this study was to investigate the CVD mortality rate trends in Lithuania over the past 18 years (2001–2018) and explore a possible link between CVD mortality rate and alcohol control policy implementation.

## Methods

The current study uses an observational design, based on yearly CVD mortality data. CVD mortality trends were investigated according to the International Classification of Diseases 10th Revision (ICD-10) codes for all CVDs (I00-I99) and three other common CVDs - ischemic heart disease (IHD) (I20-I25), cerebrovascular diseases (I60-I69) and alcoholic cardiomyopathy (ACM) (I42.6) [19]. Population mortality data for 2001–2018 were obtained from Statistics Lithuania [20] and The State Register of Death Cases and Their Causes, Institute of Hygiene [21]. Data of recorded alcohol consumed per capita (RAPC) among males and females aged 15 years and older were obtained from Statistics Lithuania [20].

### Selection of key alcohol control policies

Rehm et al. (2021) [22] have analyzed alcohol control policies implemented in Lithuania based on impact on affordability, expected immediate effect and general nature of restrictions on availability. The following five important timeline points that were identified by Rehm et al. (2021) [22] were also selected for this analysis:

1. January 1, 2008. Excise tax on alcohol beverages was increased by 10 to 20% and penalties for drunk-driving increased. TV and radio alcohol advertisements during the daytime were banned.
2. January 1, 2009. Excise tax was increased by 10 to 15%. Tax exemption for small beer breweries was canceled and nighttime (10 p.m. to 8 a.m.) takeaway alcohol sales were prohibited.
3. April 1, 2014. Excise tax was increased by 1% for ethyl alcohol and by 10 to 47% for other types of alcohol beverages.
4. March 1, 2017. Major excise tax was increased by over 111% for beer and wine, 91 to 94% for intermediate products and 23% for ethyl alcohol.
5. January 1, 2018. Legal age to purchase and to consume alcohol was increased from 18 to 20 years. Nighttime takeaway alcohol sales were prohibited from 8 p.m. to 10 a.m. on Monday-Saturday, and from 3 p.m. to 10 a.m. on Sunday. A near-total ban on alcohol advertising was introduced.

### Statistical analysis

All CVD, IHD, cerebrovascular diseases and ACM mortality rates were directly standardized by five-year age groups to the 2011–2030 European standard population [23].

Joinpoint regression analyses for all ages and a subgroup of 25–64 year-olds were performed to assess changes in the yearly sex specific mortality rates of selected diseases over the study period [24]. The Joinpoint analysis is a data-driven statistical technique that identifies bending points in data and applies various linear regression lines based on a preselected number of joining points [25]. Since there were a total 18 data points, no more than three joinpoints were identified for the analyses. Based on the maximum number of joinpoints, linear segments were fitted to the data. The Monte Carlo permutation method selects the smallest number of linear segments so that the additional junction point does not add a statistically significant linear trend [25].

For calculation of changes in mortality rates of CVD, IHD, cerebrovascular diseases and ACM, four joinpoint regression log scale models were fitted: log(*mortality*) = *β* · *year* + *α* for each period.

The first model assumed zero joinpoints. One additional joinpoint was assumed for each successive joinpoint model so that the fourth model assumed three joinpoints.

The true number of joinpoints (from 0 to 3) was determined by means of the permutation test. The estimated annual percentage change (APC) was calculated from the optimal model.

An average annual percent change (AAPC) was calculated to determine a summary measure of the 2001–2018 CVD mortality trend. The AAPC is computed as a weighted average of the APCs from the Joinpoint model, with the weights equal to the length of the APC interval.

The 95% CI was obtained in the usual manner from the standard error of the regression coefficient.

## Results

Overall, mortality rates from all CVD as well as IHD, cerebrovascular diseases and ACM decreased between 2001 and 2018 (Table 1). All CVD mortality rate decreased from 1048.6 per 100,000 population in 2001 to 749.1 per 100,000 in 2018, with an average rate of 925.6 per 100,000. The largest increase and decrease in CVD mortality rate from the previous year was found in 2005 (+ 3.8%) and 2014 (− 5.4%), respectively.

**Table 1:** Age-standardized yearly mortality rates per 100,000 people for all cardiovascular diseases, ischemic heart disease, cerebrovascular diseases and alcoholic cardiomyopathy, and annual recorded alcohol consumption rates in liters and annual percent change

Year	Cardiovascular diseases (I00-I99)		Ischemic heart disease (I20-I25)		Cerebrovascular diseases (I60-I69)		Alcoholic cardiomyopathy (I42.6)		Recorded alcohol consumption per capita	
	Cases/100,000	% change	Cases/100,000	% change	Cases/100,000	% change	Cases/100,000	% change	Value (liter)	% change
**2001**	1048.6		684.3		235.6		5.0		10.5	
**2002**	1046.3	−0.2	669.9	−2.1	244.6	3.8	4.9	− 2.0	11.1	5.7
**2003**	1025.1	−2.0	661.9	−1.2	235.2	−3.8	5.2	6.5	11.3	1.8
**2004**	1010.6	−1.4	648.1	−2.1	231.5	−1.6	5.9	13.3	12.2	8.0
**2005**	1049.5	3.8	678.3	4.7	235.6	1.8	5.8	−0.9	12.5	2.5
**2006**	1043.2	−0.6	661.0	−2.6	247.4	5.0	7.2	23.6	13.2	5.6
**2007**	1015.2	−2.7	644.3	−2.5	231.0	−6.6	9.7	34.9	13.9	5.3
**2008**	968.8	−4.6	612.6	−4.9	236.3	2.3	7.5	−23.2	13.9	0.0
**2009**	929.7	−4.0	584.6	−4.6	228.8	−3.2	4.2	−44.0	13.1	−5.8
**2010**	927.7	−0.2	602.4	3.0	222.9	−2.6	4.3	2.7	13.5	3.1
**2011**	883.0	−4.8	569.8	−5.4	216.4	−2.9	4.0	−6.9	14.7	8.9
**2012**	868.6	−1.6	566.9	−0.5	210.5	−2.7	4.6	14.9	14.7	0.0
**2013**	857.6	−1.3	559.9	− 1.2	215.0	2.1	3.4	−25.8	14.5	−1.4
**2014**	811.4	−5.4	533.4	−4.7	197.8	−8.0	3.3	−2.6	14.2	−2.1
**2015**	837.8	3.3	555.2	4.1	201.6	1.9	2.9	−11.4	14.0	−1.4
**2016**	809.6	−3.4	533.8	−3.9	195.7	−2.9	2.8	−4.8	13.2	−5.7
**2017**	778.5	−3.8	502.3	−5.9	185.1	−5.4	2.5	−12.1	12.3	−6.8
**2018**	749.1	−3.8	474.7	−5.5	182.8	−1.2	1.8	−25.1	11.2	−8.9

The IHD mortality rate decreased from 684.3 per 100,000 population in 2001 to 474.7 per 100,000 in 2018, with an average rate of 596.9 per 100,000. The largest increase and decrease in IHD mortality rate from to the previous year was found in 2005 (+ 4.7%) and 2017 (− 5.9%), respectively.

The mortality rate from cerebrovascular diseases decreased from 235.6 per 100,000 population in 2001 to 182.8 per 100,000 in 2018, with an average rate of 219.7 per 100,000. The largest increase and decrease in cerebrovascular diseases mortality rate from the previous year was found in 2006 (+ 5.0%) and 2014 (− 8.0%), respectively.

The ACM mortality rate decreased from 5.0 per 100,000 population in 2001 to 1.8 per 100,000 in 2018, with an average rate of 4.7 per 100,000. The largest increase and decrease in ACM mortality rate from to the previous year was found in 2007 (+ 67.2%) and 2018 (− 60.8%), respectively.

Between 2001 and 2018, the average RAPC in Lithuania was 13.0 l of pure alcohol per capita among those aged 15 years and older, which varied from 10.5 l in 2001 to 14.7 l in 2011 and 2012. During a period from 2001 to 2009, the RAPC increased by 32.4%, while it decreased by 23.8% from 2012 to 2018.

The Joinpoint regression analyses determined that one joinpoint, or critical year, was the ideal number of joinpoints for all CVD groups and all age groups. The results of the sex specific Joinpoint regression analyses are summarized in Table 2 for the overall age group, and in Table 3 for the 25–64 year-old age group.

**Table 2:** Trends in age-standardized mortality from cardiovascular diseases, ischemic heart disease, cerebrovascular diseases and alcoholic cardiomyopathy in all age Lithuanian men and women during 2001–2018 by the Joinpoint regression analysis

Disease code	Sex	Joinpoints (years)	Period 1		Period 2		All period (2001–2018)
			Years	APC (95% CI)	Years	APC (95% CI)	AAPC (95% CI)
**Cardiovascular diseases (I00-I99)**	Men	2006	2001–2006	0.2 (−1.0, 1.4)	2006–2018	−2.4 ^a^ (− 2.7, − 2.0)	−1.6 ^a^ (− 2.0, − 1.2)
	Women	2006	2001–2006	− 0.9 (− 2.1, 0.3)	2006–2018	−2.7 ^a^ (− 3.0, − 2.3)	−2.1 ^a^ (− 2.5, − 1.8)
**Ischemic heart disease (I20-I25)**	Men	2005	2001–2005	0.2 (− 2.0, 2.4)	2005–2018	−2.1 ^a^ (− 2.5, − 1.7)	−1.6 ^a^ (− 2.1, − 1.1)
	Women	2005	2001–2005	− 0.9 (− 3.4, 1.5)	2005–2018	− 2.3 ^a^ (− 2.8, − 1.9)	−2.0 ^a^ (− 2.6, − 1.4)
**Cerebrovascular diseases (I60-I69)**	Men	2008	2001–2008	0.0 (−1.1, 1.2)	2008–2018	− 2.0 ^a^ (− 2.7, − 1.3)	−1.2 ^a^ (− 1.7, − 0.6)
	Women	2008	2001–2008	−0.4 (− 1.5, 0.7)	2008–2018	− 2.8 ^a^ (− 3.5, − 2.1)	− 1.8 ^a^ (− 2.4, − 1.3)
**Alcoholic cardiomyopathy (I42.6)**	Men	2007	2001–2007	9.4 ^a^ (2.0, 17.2)	2007–2018	− 11.3 ^a^ (− 14.3, − 8.2)	− 4.5 ^a^ (− 7.3, − 1.6)
	Women	2007	2001–2007	10.2 (0.0, 21.4)	2007–2018	− 14.6 ^a^ (− 19.3, − 9.6)	−6.6 ^a^ (− 10.7, − 2.2)

Models are fitted on log scale: log(*mortality*) = *β* · *year* + *α* for each period. *APC* denotes annual percentage change, *AAPC* denotes average annual percentage change, *CI* denotes confidence interval. Statistics marked with ^a^ are significant at the 0.05 level.

**Table 3:** Trends in age-standardized mortality from cardiovascular diseases, ischemic heart disease, cerebrovascular diseases and alcoholic cardiomyopathy in 25–64 years’ Lithuanian men and women during 2001–2018 years by the Joinpoint regression analysis

Disease code	Sex	Joinpoints (years)	Period 1		Period 2		All period (2001–2018)
			Years	APC (95% CI)	Years	APC (95% CI)	AAPC (95% CI)
**Cardiovascular diseases (I00-I99)**	Men	2006	2001–2006	4.8^a^ (1.9, 7.8)	2006–2018	−4.3 ^a^ (− 5.0, − 3.5)	−1.7 ^a^ (− 2.6, − 0.8)
	Women	2006	2001–2006	3.9 ^a^ (2.2, 5.7)	2006–2018	−5.2 ^a^ (− 5.7, − 4.8)	− 2.6 ^a^ (− 3.2, − 2.1)
**Ischemic heart disease (I20-I25)**	Men	2006	2001–2006	3.6 ^a^ (0.8, 6.6)	2006–2018	− 4.1 ^a^ (− 4.9, − 3.3)	−1.9 ^a^ (− 2.8, − 1.0)
	Women	2006	2001–2006	5.4 ^a^ (2.6, 8.4)	2006–2018	− 5.2 ^a^ (− 5.9, − 4.4)	− 2.2 ^a^ (− 3.0, − 1.3)
**Cerebrovascular diseases** **(I60-I69)**	Men	2007	2001–2007	2.1 (− 0.9, 5.3)	2007–2018	− 3.9 ^a^ (− 5.2, − 2.6)	−1.8 ^a^ (− 3.0, − 0.6)
	Women	2010	2001–2010	− 1.2 (− 3.1, 0.8)	2010–2018	− 6.6 ^a^ (− 9.2, − 3.9)	− 3.8 ^a^ (− 5.2, − 2.3)
**Alcoholic cardiomyopathy (I42.6)**	Men	2007	2001–2007	10.5 ^a^ (2.6, 19.0)	2007–2018	− 11.3 ^a^ (− 14.4, − 8.1)	−4.1 ^a^ (− 7.1, − 1.1)
	Women	2007	2001–2007	12.2 ^a^ (1.2, 24.4)	2007–2018	− 15.1 ^a^ (− 20.0, − 9.8)	− 6.3 ^a^ (− 10.7, − 1.7)

Models are fitted on log scale: log(*mortality*) = *β* · *year* + *α* for each period. *APC* denotes annual percentage change, *AAPC* denotes average annual percentage change, *CI* denotes confidence interval. Statistics marked with ^a^ are significant at the 0.05 level.

**Fig. 1:**
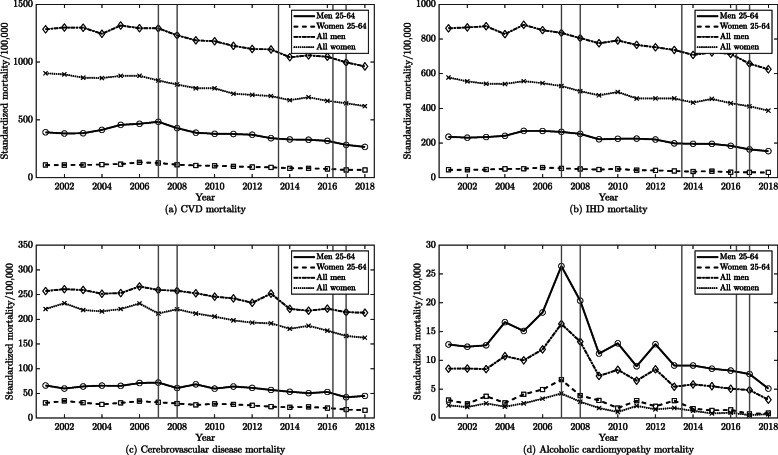
The trends in age-standardized mortality rates from cardiovascular diseases, ischemic heart diseases, cerebrovascular diseases and alcoholic cardiomyopathy per 100,000 people among Lithuanian men and women in all ages and in 25–64 years’ during 2001–2018. Note: Vertical lines illustrate time points of the five alcohol policies investigated in this analysis. As the mortality trends describes the end of the year state, each of the timeline policy points was visualized by setting it 1 year earlier with an aim to indicate an end of the year line before the initiation of a specific policy

The CVD mortality rates significantly decreased on average by 1.6% (95% CI -2.0; − 1.2) among men and by 2.1% (95% CI -2.5; − 1.8) among women per year (Fig. 1a). 2006 was identified as the critical year for all CVD. The CVD mortality rates did not change significantly for both men and women from 2001 to 2006. However, significant decreases in CVD mortality rates were observed from 2006 to 2018 for both men and women by an average of 2.4% (95% CI -2.7; − 2.0) and 2.7% (95% CI -3.0; − 2.3), respectively.

The mortality rates from IHD, cerebrovascular diseases and ACM significantly decreased for both men and women between 2001 and 2018. The IHD, cerebrovascular diseases and ACM mortality rates decreased each year by an average of 1.6% (95% CI -2.1; − 1.1), 1.2% (95 CI -1.7; − 0.6) and 4.5% (95% CI -7.3; − 1.6) among men and by 2.0% (95% CI -2.6; − 1.4), 1.8% (95% CI 2.4; − 1.3) and 6.6% (95% CI -10.7; − 2.2) among women, respectively (Fig. 1b, c and d). The critical year points for IHD, cerebrovascular diseases and ACM were respectively 2005, 2008 and 2007. The mortality rates for IHD, cerebrovascular diseases and ACM significantly decreased after their respective critical year points (Fig. 1b and c). IHD mortality rate decreased each year after 2005 by an average of 2.1% (95% CI -2.5; − 1.7) among men and 2.3% (95% CI -2.8; − 1.9) among women, and cerebrovascular diseases mortality rate decreased each year after 2008 by an average of 2.0% (95% CI -2.7; − 1.3) among men and 2.8% (95% CI -3.5; − 2.1) among women. The mortality rate for ACM increased significantly between 2001 and 2007 among men by an average of 9.4% (95% CI 2.0; 17.2) per year but significant changes were not observed among women for the same time period. However, ACM mortality rate decreased significantly after 2007 among men and women by an average of 11.3% (95% CI -14.3; − 8.2) and 14.6% (95% CI -19.3; − 9.6), respectively.

Among 25–64 year-old men and women, CVD mortality decreased significantly on average by 1.7% (95% CI -2.6; − 0.8) and 2.6% (95% CI -3.2; − 2.1) per year, respectively, from 2001 to 2018 (Fig. 1a). 2006 was identified as the critical year for all CVD among the 25–64 year-old population. CVD mortality rates increased significantly between 2001 to 2006 by 4.8% (95% CI 1.9; 7.8) in men and by 3.9% (95% CI 2.2; 5.7) in women, but significantly decreased on average by 4.3% (95% CI -5.0; − 3.5) and 5.2% (95% CI -5.7; − 4.8), respectively, between 2006 and 2018 (Table 3).

Between 2001 and 2018, mortality from IHD among both men and women aged 25 to 64 years decreased significantly on average by 1.9% (95% CI -2.8; − 1.0) and 2.2% (95% CI -3.0; − 1.3) per year, respectively (Fig. 1b). 2006 was identified as the critical year for IHD among the 25 to 64 year-old population. Mortality from IHD increased significantly between 2001 and 2006 among men and women by 3.6% (95% CI 0.8; 6.6) and 5.4% (95% CI 2.6; 8.4) per year, respectively, and decreased significantly on average by 4.1% (95% CI -4.9; − 3.3) and 5.2% (95% CI -5.9; − 4.4), respectively, from 2006 to 2018.

Between 2001 and 2018, mortality from cerebrovascular diseases among both men and women aged 25 to 64 years decreased significantly on average by 1.8% (95% CI -3.0; − 0.6) and 3.8% (95% CI -5.2; − 2.3) per year, respectively (Fig. 1c). 2007 and 2010 were identified as the critical year for cerebrovascular diseases among 25 to 64 year-old men and women, respectively. Mortality from cerebrovascular diseases trends among men and women 25 to 64 years old were without significant changes until their respective joinpoints, after which cerebrovascular diseases mortality decreased significantly by an average of 3.9% (95% CI -5.2; − 2.6) and 6.6% (95% CI -9.2; − 3.9) per year, respectively.

Between 2001 and 2018, mortality from ACM among men and women aged 25 to 64 years decreased significantly on average by 4.1% (95% CI -7.1; − 1.1) and 6.3% (95% CI -10.7; − 1.7) per year, respectively (Fig. 1d). 2007 was identified as the critical year for ACM among the 25 to 64 year-old population. Mortality from ACM increased significantly between 2001 and 2007 among both men and women by an average of 10.5% (95% CI 2.6; 19.0) and 12.2% (95% CI 1.2; 24.4), respectively. The ACM mortality rate for men and women then decreased significantly from 2007 to 2018 by an average of 11.3% (95% CI -14.4; − 8.1) and 15.1% (95% CI -20.0; − 9.8), respectively.

Figure 1 shows that changes in the mortality rates trends for all CVDs, IHD, cerebrovascular diseases and especially ACM coincided with the alcohol policies enacted on the January 1, 2008, January 1, 2009, April 1, 2014 and March 1, 2017 in Lithuanian.

## Discussion

Our study determined the age-standardized mortality rates from 2001 to 2018 for all CVD, IHD, cerebrovascular diseases and ACM to identify any potential links between the implementation of selected most effective alcohol control policies and changes in CVD mortality rates among Lithuanians. All CVD mortality significantly decreased from 2001 to 2018 in both sexes in Lithuania, however the declining trends were more prominent in women than in men. We identified 2005, 2006, 2007, 2008 and 2010 as critical years in CVD mortality rate trends, after which a significant decrease was observed.

The largest AAPC increase (+ 3.8%) in all CVD mortality rates was found between 2004 and 2005, among all ages. As described by Miščikienė et al. (2020) [18], alcohol policies on taxation and advertising were loosened in 2001 and 2002, and further loosened in 2004 as part of legal harmonization with the European Union. This could have contributed to the increased affordability of alcohol beverages and consequently higher alcohol attributable burden.

Among the different CVD groups investigated, the changes in ACM mortality rate trends coincided best with the selected alcohol policy enactment dates. Direct link was observed when analyzing mortality from cerebrovascular diseases in the study population; mortality trends started to decrease significantly in average per year by 2.0% in men and by 2.8% in women after the implementation of alcohol control policies in 2008.

The analysis suggests that there might be a close relation between the implemented alcohol control policies and ACM mortality (especially in men), which is consistent with findings from Russia, a country with a similar experience in the field of alcohol control as Lithuania [26].

As we cannot exclude the supporting effects of the alcohol control policies on the overall declining trends in CVD, we cannot conclude that there was an effect either. The alcohol control policies which were anticipated to be highly impactful did not coincide with rapid declines of CVD mortality in women and men aged at least 15 years or among the 25 to 64 years-old population.

The misalignment between the critical year points identified in this study and the policy implementation time points may be partly explained by recent improvements in the CVD risk profile of Lithuanians, which may have also contributed to the decreased mortality rates between 2001 and 2018.

During the analyzed period, many changes occurred in the CVD risk profile of the Lithuanian population. In 2006, the National Programme for the Screening and Preventive Management of the High Cardiovascular Risk Individuals (40–54 years-old men and 50–64 years-old women) was launched in Lithuania and the prevalence of CVD risk factors has reduced since. The percentage of smoking cessation has increased significantly and a decreasing trend in smoking prevalence, especially among men was estimated [27, 28]. The prevalence of dyslipidemia and arterial hypertension has declined [29, 30], and a significant reduction in the prevalence of metabolic syndrome was identified among women [31]. The previous studies have confirmed that CVD prevention programs can reduce CVD mortality in the long term [32–34].

Furthermore, significant efforts have been made during the last two decades to improve the diagnosis, management, and treatment of acute myocardial infarction, including pre-hospital and treatment procedures during hospitalization and outpatient care [35, 36]. Nowadays, cardiac reperfusion rates used as a measure of the effectiveness of acute myocardial infarction treatment in Lithuanian hospitals are similar to those in Western and Northern European countries [37–39]. Also, the clinical diagnosis of patients with CVD has improved due to the application of the newest examination methodologies and diagnostic tools. All the above-mentioned measures could have played a role in a declining trend in CVD mortality.

Long-term behavioral changes were also observed during 2001 to 2018 in the Lithuanian population. Nutrition habits have improved: the consumption of animal fat declined, while the use of vegetable oil and consumption of fresh vegetables increased [40]. Based on the Health Interview Survey carried out by Statistics Lithuania, the prevalence of daily smoking in men decreased from 42.1% in 2005 to 29.9% in 2019 and remained stable in women (9.8% in 2005 and 9.7% in 2019) [41].

### Study limitations

Even though this study analyzed a relatively long period of time, it was subject to limitations. First, the study design controlled for underlying seasonal patterns and secular trends, but the use of ecological data can be considered as limitation. Second, it was impossible to make a proper full assessment of the possible effects of all other relevant confounding factors linked to observed trends and decrease in alcohol consumption. Thus, in any ecological analysis alternative explanations must be considered. Finally, we suspect under-reporting of alcohol-related death in death certificates, including alcoholic cardiomyopathy, due to high stigma related to the heavy drinking culture in Lithuania.

## Conclusions

Our study showed that all CVD mortality, including IHD, cerebrovascular diseases and ACM, has declined in Lithuania over the past two decades, and declining trends were more prominent in women than in men, especially among individuals 25–64 years of age. Among the different CVD groups investigated, the onset of the declining ACM mortality rate trend coincided best with the selected alcohol policy enactment dates. Future studies are needed to estimate the impact of the alcohol control policies implemented in Lithuania on CVD mortality rates using more targeted statistical methods.

## Data Availability

The datasets used and/or analysed during the current study are available from the corresponding author on reasonable request.

## Abbreviations

AAPC: An average annual percent change
APC: Annual percentage change
ACM: Alcoholic cardiomyopathy
CI: Confidence interval
CVD: Cardiovascular diseases
ICD-10: International Classification of Diseases 10th Revision
IHD: Ischemic heart diseases
RAPC: Recorded alcohol consumed per capita
